# KEAP1 and done? Targeting the NRF2 pathway with sulforaphane

**DOI:** 10.1016/j.tifs.2017.02.002

**Published:** 2017-11

**Authors:** Albena T. Dinkova-Kostova, Jed W. Fahey, Rumen V. Kostov, Thomas W. Kensler

**Affiliations:** a Jacqui Wood Cancer Centre, Division of Cancer Research, School of Medicine, University of Dundee, Dundee, DD1 9SY, Scotland, UK; b Lewis B. and Dorothy Cullman Chemoprotection Center, Department of Pharmacology and Molecular Sciences, Johns Hopkins University School of Medicine, Baltimore, MD 21205, USA; c Division of Clinical Pharmacology, Department of Medicine, Johns Hopkins University School of Medicine, Baltimore, MD 21205, USA; d Center for Human Nutrition, Department of International Health, Johns Hopkins University Bloomberg School of Public Health, Baltimore, MD 21205, USA; e Department of Pharmacology & Chemical Biology, University of Pittsburgh, Pittsburgh, PA 15261, USA

**Keywords:** Sulforaphane, KEAP1, NRF2, Cytoprotection, Clinical trial

## Abstract

**Background:**

Since the re-discovery of sulforaphane in 1992 and the recognition of the bioactivity of this phytochemical, many studies have examined its mode of action in cells, animals and humans. Broccoli, especially as young sprouts, is a rich source of sulforaphane and broccoli-based preparations are now used in clinical studies probing efficacy in health preservation and disease mitigation. Many putative cellular targets are affected by sulforaphane although only one, KEAP1-NRF2 signaling, can be considered a validated target at this time. The transcription factor NRF2 is a master regulator of cell survival responses to endogenous and exogenous stressors.

**Scope and Approach:**

This review summarizes the chemical biology of sulforaphane as an inducer of NRF2 signaling and efficacy as an inhibitor of carcinogenesis. It also provides a summary of the current findings from clinical trials using a suite of broccoli sprout preparations on a series of short-term endpoints reflecting a diversity of molecular actions.

**Key Findings and Conclusions:**

Sulforaphane, as a pure chemical, protects against chemical-induced skin, oral, stomach, colon, lung and bladder carcinogenesis and in genetic models of colon and prostate carcinogenesis. In many of these settings the antitumorigenic efficacy of sulforaphane is dampened in *Nrf2*-disrupted animals. Broccoli preparations rich in glucoraphanin or sulforaphane exert demonstrable pharmacodynamic action in over a score of clinical trials. Measures of NRF2 pathway response and function are serving as guideposts for the optimization of dose, schedule and formulation as clinical trials with broccoli-based preparations become more commonplace and more rigorous in design and implementation.

## Highlights

• Sulforaphane, an isothioicyante found in cruciferous vegetables, activates the KEAP1-NRF2 cell survival response.
• Sulforaphane inhibits carcinogenesis in many animal models; in some cases *Nrf2* knockout mice are refractory to protection.
• Broccoli preparations rich in sulforaphane have been used in clinical trials examining health preservation and disease mitigation.
• Many of these trials utilize pharmacodynamics biomarkers reflective of altered NRF2 signaling, although other markers are also under study.

## 1 Introduction

Development of proactive prevention programs, e.g., preventive, predictive, personalized and participatory (“P4”), are emerging as important elements to control a number of chronic, degenerative diseases. The transformation of cancer prevention through personalized or precision medicine is a prime example of current opportunity (Kensler et al., 2016), although any one of the P4 elements alone will not be sufficient. Within the context of cancer, much of the expanding global burden will occur in the developing and recently developed countries. Many in these regions, and arguably most regions, will have neither access nor ability to afford the latest generation of molecular-targeted pharmaceuticals. By contrast, implementation programs for tobacco control, vaccination, screening, as well as public health programs promoting physical activity and consumption of healthier diets will have greater impact on population health broadly and cancer prevention specifically. Access to local foodstuffs containing bioactive phytochemicals may offer a frugal or “green” (Fahey, Talalay, & Kensler, 2012a) means for accelerating disease prevention. Appreciation of the mechanisms of the action of such phytochemicals will facilitate the utilization of indigenous protective foods or perhaps guide the introduction of culturally appropriate new foods into their diets.

**Fig. 1:**
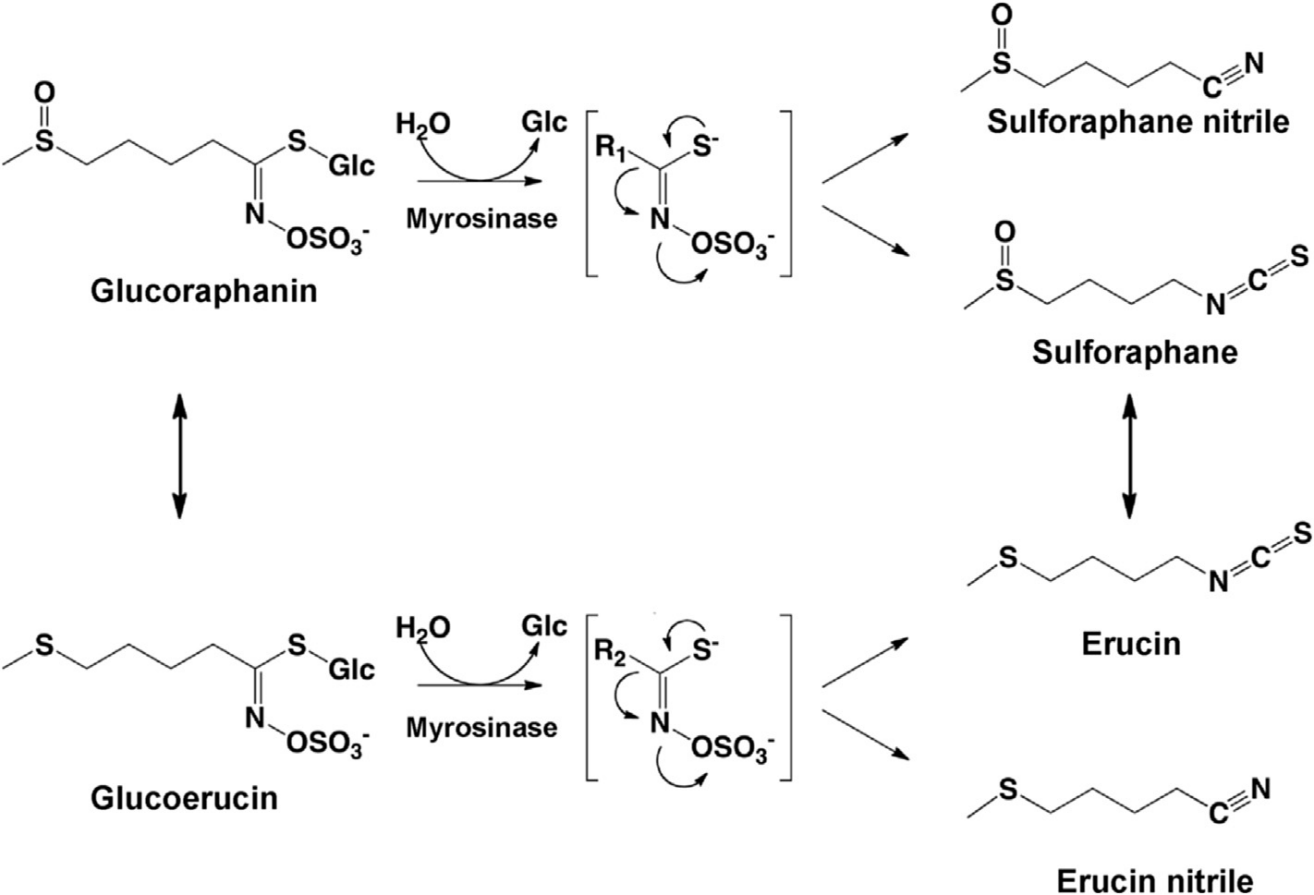
**The myrosinase reaction and the interconversion of sulforaphane and erucin.** The glucosinolates glucoraphanin and glucoerucin are hydrolyzed by β-thioglucosidases (myrosinases) to give unstable aglycones and liberate glucose. Depending on the reaction conditions, a variety of reactive products can be formed, the most common of which are the isothiocyanates (sulforaphane and erucin) and their corresponding nitriles. In mammals, glucoraphanin is also taken up from the gut to the liver where it is interconverted to its reduced analog, glucoerucin, as is sulforaphane to erucin. R_1_ = 4-(methylsulfinyl)butane; R_2_ = 4-(methylthio)butane.

Based on findings from epidemiology studies suggesting that frequent consumption of cruciferous vegetables was associated with lower incidence of multiple tumor types, Talalay and colleagues screened extracts of these and other vegetables for bioactive molecules for efficacy and potency in the induction of enzymes known to detoxify carcinogens (Zhang, Kensler, Cho, Talalay & Posner, 1992), a process now known to involve NRF2 signaling. Sulforaphane [1-isothiocyanato-4-(methylsulfinyl)butane] (Fig. 1) a phytochemical belonging to a large chemical family of isothiocyanates was thus identified. Sulforaphane is formed from the stable, water-soluble precursor glucosinolate termed glucoraphanin in a variety of cruciferous vegetables including broccoli, Brussels sprouts, cauliflower, and cabbage by myrosinase, a β-thioglucoside glucohydrolase (EC 3.2.1.147), during damage of plant integrity or by hydrolysis by uncharacterized β-thioglucosidases of the gut microflora (Shapiro, Fahey, Wade, Stephenson, & Talalay, 2001). During glucoraphanin hydrolysis, glucose is liberated and an unstable aglycone is formed that spontaneously rearranges to metabolites such as sulforaphane (Fig. 1). At high or neutral pH, sulforaphane will be the primary product of glucoraphanin hydrolysis. In contrast, at acidic pH, or in the presence of Fe^2+^, with the enzyme epithiospecifier protein, the production of a nitrile, which is less bioactive, will be favored (Hayes, Kelleher, & Eggleston, 2008). In mammals, glucoraphanin is also taken up from the gut to the liver where it is interconverted to its reduced glucosinolate analog, glucoerucin, as is sulforaphane to its corresponding reduced isothiocyanate analog, erucin [1-isothiocyanato-4-(methylthio)butane] (Bheemreddy and Jeffery, 2007, Melchini and Traka, 2010). The highest concentrations of glucosinolates are typically found in reproductive organs of the plant, including dormant and germinating seeds, and developing inflorescences, followed by young leaves, roots, and mature leaves, which is consistent with the function of glucosinolate-myrosinase system as defensive mechanism in the plant (Brown, Tokuhisa, Reichelt, & Gershenzon, 2003). Three-day-old broccoli sprouts contain 10–100 times higher levels of glucoraphanin than do mature broccoli (Fahey, Zhang, & Talalay, 1997). With the virtues of, safety, effectiveness, feasibility and low cost, sulforaphane in the milieu of broccoli (especially broccoli sprout and seed preparations) has attracted extensive interest as a potential preventive agent in humans.

The pharmacokinetics of sulforaphane and glucoraphanin, as either pure phytochemical studied in animals (Cornblatt et al., 2007, Hu et al., 2004), or in a variety of plant matrices in clinical studies (Egner et al., 2011, Fahey et al., 2012b, Fahey et al., 2016, Shapiro et al., 2001), have been well characterized. Sulforaphane is readily absorbed in humans and is rapidly eliminated. Upwards of 70% of an administered dose of sulforaphane can be recovered as thiol conjugates in the urine; the biological half-life is only a few hours. By contrast, glucoraphanin has poor bioavailability, with only about 10% of an administered dose being recovered as thiol conjugates of sulforaphane in urine. The elimination phase is also longer, reflecting a poor, slow and highly variable conversion of the glucosinolate to isothiocyanate in the absence of plant myrosinase. As a consequence, recent preparations for use in clinical studies feature both plant based sources for glucoraphanin and myrosinase (Fahey et al., 2015). Despite a near quarter century since the (re)discovery of sulforaphane (Zhang et al., 1992), studies on the pharmacodynamic actions of sulforaphane in humans have been quite limited. This point stands in stark contrast to the many hundreds of publications probing mechanisms of action in cell culture and animal models. As reviewed elsewhere, dozens of targets and pathways have been identified as potential mediators of the chemoprotective actions of sulforaphane (Brown and Hampton, 2011, Hayes et al., 2008, Zhang, 2012): few have undergone serious validation. Two key approaches for target validation arise from the questions: Does genetic disruption of the target alter sensitivity to carcinogenesis or other disease states in animal models? Does genetic disruption of the target abolish or attenuate the chemopreventive efficacy of candidate agents, such as sulforaphane? While not dismissing many of these actions as of limited importance, the NRF2 pathway, as detailed in this review, stands alone as a validated target for the activity of sulforaphane. Although unlikely to be of unilateral importance, measures of pathway response and function can serve as guideposts for the optimization of dose, schedule and formulation as clinical trials with broccoli-based preparations become more commonplace and more rigorous in design and implementation.

## 2 KEAP1-NRF2 signaling: a molecular target for sulforaphane

**Fig. 2:**
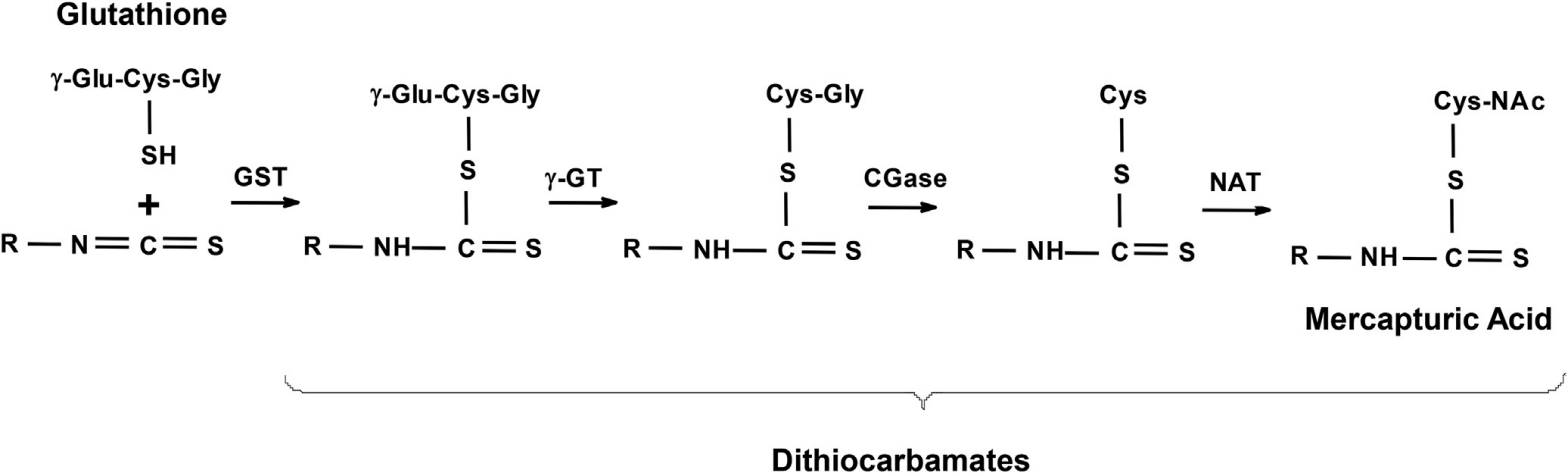
**Metabolism of isothiocyanates in mammalian cells.** The central carbon of the isothiocyanate (—N=C=S) group is electrophilic and reacts readily with sulfur-, nitrogen-, and oxygen-centered nucleophiles. The most common reaction in mammalian cells is conjugation with sulfhydryl groups, such as the sulfhydryl group of cysteine in proteins and glutathione. The reaction with glutathione is catalyzed by glutathione *S*-transferases (GSTs), and the resulting product is cleaved sequentially by γ-glutamyl-transpeptidase (γ-GT), cysteinyl-glycinease (GCase), and *N*-acetyltransferase (NAT) to give the *N*-acetylcysteine conjugate (mercapturic acid). The conjugates are collectively known as dithiocarbamates.

The most characteristic feature of sulforaphane is its high chemical reactivity due to the electrophilicity of the central carbon of the isothiocyanate (—N=C=S) group. The isothiocyanate group reacts readily with sulfur-, nitrogen-, and oxygen-centered nucleophiles [reviewed in (Mi et al., 2011, Zhang, 2012)]. Most common in cells is the reversible reaction of isothiocyanates with cysteine residues in proteins and glutathione, leading to the formation of thiocarbamate products, which are subsequently metabolized by the mercapturic acid pathway (Fig. 2). Irreversible alkylation reactions of isothiocyanates with the α-amino groups in N-terminal residues of proteins, with the ε-amino groups of lysine, or even with secondary amines, such as proline, are also possible, and the products of these reactions are known as thioureas (Kumar and Sabbioni, 2010, Nakamura et al., 2009). In theory, the isothiocyanates can also react with hydroxyl group-containing amino acid residues (e.g., tyrosine), although this probably does not occur under physiological conditions.

***Targeting KEAP1.*** Cysteine residues with low pKa values are especially reactive with isothiocyanates. At physiological pH, such cysteines exist as thiolate anions that are primed for nucleophilic attack on the electrophilic substrate. Upon entry into the cell, sulforaphane chemically reacts with Kelch-like ECH associated protein 1 (KEAP1) (Itoh et al., 1999), a protein endowed with a number of reactive cysteine residues which function as sensors for numerous oxidants and electrophiles (termed inducers), including the isothiocyanates (Dinkova-Kostova et al., 2005, Dinkova-Kostova et al., 2016). KEAP1 is a dimeric multidomain 624-amino acid protein that serves as a substrate adaptor for a Cullin3-based Cullin-RING E3 ubiquitin ligase (CRL) multisubunit protein complex. Based on its amino acid sequence, KEAP1 has five distinct domains: (i) an N-terminal region (NTR, amino acids 1–49), (ii) a Broad complex, Tramtrack, and Bric à brac (BTB) domain (amino acids 50–179), through which KEAP1 forms a homodimer and also interacts with Cullin3, (iii) an intervening region (IVR, also known as BACK domain, amino acids 180–314), which is especially cysteine-rich and contains 8 cysteine residues among its 134 amino acids, (iv) a Kelch domain, comprising six Kelch motifs (amino acids 315–359, 361–410, 412–457, 459–504, 506–551, and 553–598), through which KEAP1 binds to its substrates, and (v) a C-terminal region (CTR, amino acids 599–624). Although there is currently no crystal structure of the full-length KEAP1 protein, molecular modeling (Fourquet et al., 2010, McMahon et al., 2010, Quinti et al., 2016) and multiple crystal structures of the individual BTB (Cleasby et al., 2014, Huerta et al., 2016) and Kelch (Beamer et al., 2005, Fukutomi et al., 2014, Komatsu et al., 2010, Li et al., 2004, Padmanabhan et al., 2005) domains of KEAP1, together with a reconstituted single particle electron microscopy structure (Ogura et al., 2010) have provided valuable structural information on KEAP1 and the way by which it interacts with its binding partners.

**Fig. 3:**
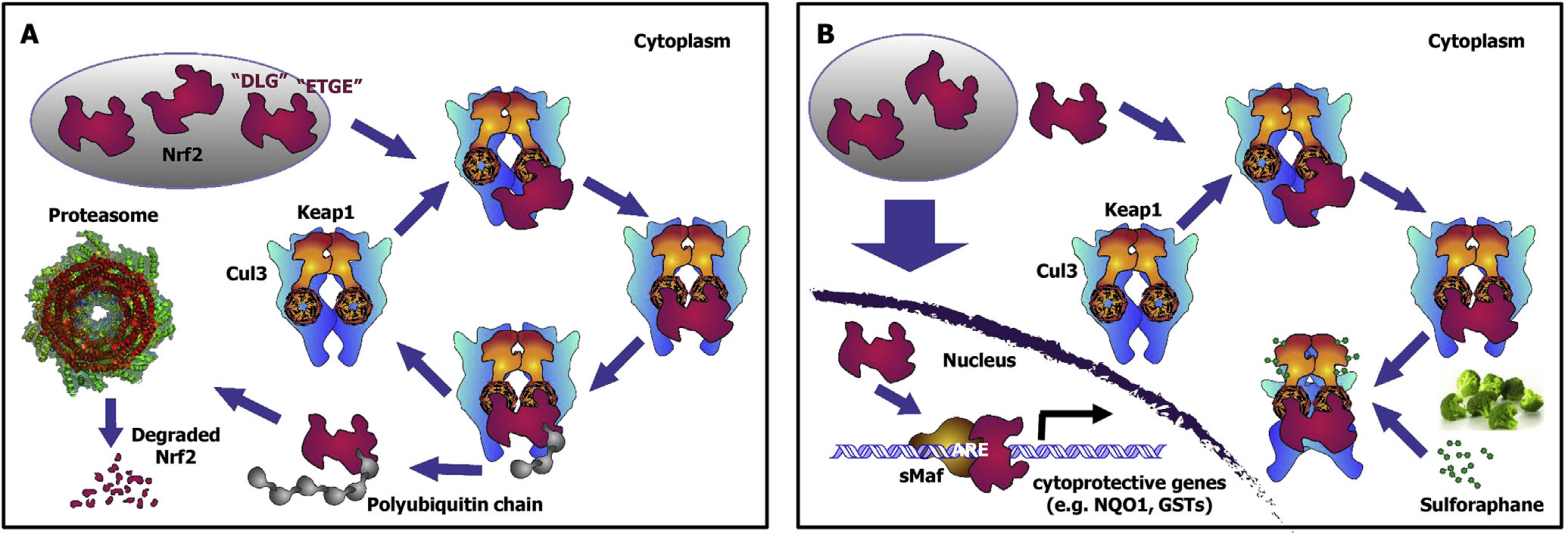
**The cyclical model of KEAP1-mediated degradation of NRF2. (A)** At homeostatic conditions, de novo synthesized NRF2 binds sequentially to the Kelch domains of the KEAP1 dimer, first through its high affinity “ETGE” binding motif followed by the low affinity “DLG” binding motif. Fully bound NRF2 is ubiquitinated and degraded through the proteasome. Free KEAP1 is regenerated. **(B)** Sulforaphane blocks the cycle by chemically modifying cysteine sensor(s) of KEAP1 and disabling its substrate adaptor function. Consequently, NRF2 is not degraded, KEAP1 is not regenerated, de novo synthesized NRF2 accumulates and, as a heterodimer with a small Maf transcription factor (sMaf), initiates transcription of target genes.

***KEAP1 Substrate.*** The best-characterized substrate of KEAP1 is transcription factor NF-E2 p45-related factor 2 (NRF2) (Itoh et al., 1997, Itoh et al., 1999). At homeostatic conditions, KEAP1 targets NRF2 for ubiquitination and proteasomal degradation (Cullinan et al., 2004, Kobayashi et al., 2004, Zhang et al., 2004). Using a mechanism known as “hinge-and-latch” (Tong et al., 2007), one molecule of NRF2 binds to the KEAP1 dimer via two distinct motifs residing in the N-terminal Neh2 domain of the transcription factor. These are known as the “DLG” and the “ETGE” motifs, which are situated at either side of a central lysine-rich α-helix. The affinity for the ETGE motif is 200-fold greater than that for the DLG motif, and the ETGE motif is thought to function as the “hinge”, whereas the DLG motif functions as the “latch”, positioning the NRF2 lysine-rich helix for ubiquitination (McMahon et al., 2006, Tong et al., 2006). The “DLG” and the “ETGE” motifs form β-turn structures which bind via electrostatic interactions between their acidic aspartate and glutamate residues with arginine residues 380, 415, and 483 in the Kelch domain of KEAP1. Binding to both motifs is essential for the KEAP1-mediated ubiquitination of NRF2 (McMahon et al., 2006) that occurs via a highly efficient cyclic mechanism (Fig. 3), in which KEAP1 is continuously regenerated (Baird, Lleres, Swift, & Dinkova-Kostova, 2013). Chemical modification of the sensor cysteines of KEAP1 by inducers, such as sulforaphane, blocks the cycle of KEAP1-dependent NRF2 degradation. This block allows *de novo* synthesized NRF2 to accumulate, translocate to the nucleus, and initiate transcription of its downstream target genes.

***Modifying KEAP1 Cysteines.*** By use of UV-VIS spectroscopy cysteine modifications within KEAP1 were shown to occur when the recombinant murine protein was incubated with sulforaphane (Dinkova-Kostova et al., 2002). By use of mutagenesis analysis, Zhang and Hannink found that ectopically-expressed KEAP1 in which C151 in the BTB domain was mutated to a serine is able to repress NRF2 even upon sulforaphane treatment, thus implicating C151 as one of the cysteines which is specifically responsive to sulforaphane (Zhang & Hannink, 2003). Over the subsequent years, it became clear that C151 is one of the most reactive and critical cysteines in KEAP1 for NRF2 signaling. McMahon and Hayes confirmed C151 as a target for sulforaphane by use of the biotin-switch technique (McMahon et al., 2010). Additionally, molecular modeling and mutagenesis experiments further demonstrated that C151 is particularly highly reactive as it is spatially surrounded by basic amino acids (H129, K131, R135, K150, and H154) which facilitate electrophilic addition to C151. Indeed, a mutant of KEAP1 in which K131, R135, and K150 were replaced by methionine residues had a greatly reduced sensor activity. A molecular model by Fourquet and Toledano predicted that C151 is remotely positioned from both the BTB dimerization interface and Cullin3, and also implicated the basic amino acid environment in the increased reactivity of this cysteine (Fourquet et al., 2010). Based on mutagenesis analysis, Mesecar proposed a model whereby large residues at position 151 cause steric clashes that lead to alteration of the KEAP1-Cullin3 interaction, ultimately resulting in impaired ability of KEAP1 to target NRF2 for ubiquitination (Eggler, Small, Hannink, & Mesecar, 2009), although a crystal structure of the KEAP1 C151W mutant BTB domain showed no obvious changes that would impact Cullin3 binding (Cleasby et al., 2014). Mass-spectrometry approaches have shown that, depending on the experimental conditions, in addition to C151, sulforaphane can also modify other cysteines within KEAP1, including cysteines residing in the Kelch domain (Eggler et al., 2007, Hong et al., 2005, Hu et al., 2011). The importance of C151 in the molecular actions of sulforaphane *in vivo* was cemented by Yamamoto and colleagues (Takaya et al., 2012) who generated KEAP1-C151 expressing cells from genetically engineered mice, sulforaphane evoked only marginal inductive responses in the C151 mutant cells compared to wild-type; nuclear translocation of NRF2 and induction of its target genes (*Gclc, Nqo1*) were impeded by >75%. Of note, although C151 is the main sensor cysteine for sulforaphane, KEAP1 has other reactive cysteine residues, which sense specific types of inducers (reviewed in Dinkova-Kostova et al., 2016, Hayes and Dinkova-Kostova, 2014). Thus C273 and C288 are modified by inducers such as 4-hydroxynonenal and cyclopentenone prostaglandins, whereas C434 is modified by 8-nitro-cGMP. C226 and C613 form the sensor for metals, hydrogen peroxide and hydrogen sulfide.

***Exogenous Modifiers of KEAP1 and Target Genes.*** Chemical modification of the sensor cysteine(s) of KEAP1 (by sulforaphane and other inducers) impairs its substrate adaptor function, leading to NRF2 accumulation and enhanced transcription of NRF2-dependent genes. These genes have antioxidant response elements (AREs) in their upstream regulatory regions [reviewed in (Nguyen et al., 2009, Tebay et al., 2015)], which are the sites of binding of NRF2 as a heterodimer with a small Maf transcription factor [reviewed in (Katsuoka & Yamamoto, 2016)]. The use of high-throughput chromatin-immunoprecipitation with parallel sequencing methodology identified more than 600 NRF2-target genes (Malhotra et al., 2010). NRF2-dependent genes encode multiple functionally diverse enzymes and other proteins with cytoprotective activities [reviewed in (Hayes and Dinkova-Kostova, 2014, Kensler et al., 2007)]. These include: antioxidant enzymes (e.g., heme oxygenase 1, NAD(P)H:quinone oxidoreductase 1, thioredoxin, thioredoxin reductase, as well as enzymes that participate in the synthesis and regeneration of glutathione, such as the catalytic and regulatory subunits of γ-glutamylcysteine ligase, glutathione reductase); conjugating enzymes (e.g., glutathione S-transferases); proteins that enhance the export of xenobiotics and/or their metabolites (e.g., solute carrier- and ATP-binding cassette transporters); enzymes that promote the synthesis of reducing equivalents, i.e., NADPH (e.g., glucose 6-phosphate dehydrogenase, 6-phosphogluconate dehydrogenase, malic enzyme 1, isocitrate dehydrogenase 1); enzymes that inhibit inflammation (e.g., leukotriene B_4_ dehydrogenase); proteins that protect against iron overload (e.g., ferritin, metallothionein); proteins that participate in the repair and removal of damaged proteins (e.g., subunits of the 26S proteosome) and organelles (e.g., autophagy-related proteins such as SQSTM1/p62, ULK1 and ATG5). In addition, NRF2 engages in crosstalk with other transcription factors, such as the aryl hydrocarbon receptor (AhR) (Shin et al., 2007, Yeager et al., 2009), the retinoic X receptor alpha (RXRα) (Wang et al., 2013), NF-kB (Nair, Doh, Chan, Kong, & Cai, 2008), p53 (Chen et al., 2012), Notch1 (Wakabayashi et al., 2015, Wakabayashi et al., 2010a, Wakabayashi et al., 2010b) and heat shock factor 1 (Hsf1) (Dayalan Naidu, Kostov, & Dinkova-Kostova, 2015), thus influencing indirectly the expression of their respective target genes.

***Nrf2 Stress Response.*** The networks of these NRF2-directed transcriptional programs allow the cell to adapt and survive under various conditions of stress and are at the heart of the chemoprotective effects of NRF2 signaling. Cellular protection also requires alterations in metabolism and bioenergetics, and although the underlying mechanisms are not well understood, it is becoming increasingly clear that NRF2 activation has a profound effect on mitochondrial function and intermediary metabolism [reviewed in (Dinkova-Kostova and Abramov, 2015, Hayes and Dinkova-Kostova, 2014)]. Another prominent feature of NRF2 activation with high relevance to chemoprotection is inhibition of inflammation. Chronic inflammation and oxidative stress are the underlying causes for most of the common human pathologies, including cardiovascular and neurodegenerative disease, as well as cancer (Liby & Sporn, 2012). It is the ability to suppress simultaneously oxidative stress and inflammation (processes that accompany each other and if persistent, often have deleterious effects) that makes the activation of NRF2 signaling such a powerful and efficient protector. The broad antioxidant effects of NRF2 are largely due to its direct transcriptional targets, which as explained earlier, include enzymes with antioxidant activities. The anti-inflammatory activities of NRF2 are more complex and include transcriptional upregulation of enzymes encoded by NRF2-target genes, such as leukotriene B_4_ dehydrogenase (Dick et al., 2001, Primiano et al., 1998), but also suppression of the expression of genes encoding major pro-inflammatory cytokines, such as IL-6 and IL-1β (Knatko et al., 2015; Kobayashi et al., 2016). Finally, excessive oxidative stress and inflammation can cause irreversible damage to proteins and organelles, and NRF2 activation facilitates their clearance by regulating the expression of genes encoding multiple proteasomal subunits (Kwak and Kensler, 2006, Kwak et al., 2003) and autophagy-related proteins (Pajares et al., 2016). In addition to direct anti-inflammatory effects mediated through NRF2 signaling, sulforaphane may impair the redox-sensitive DNA binding and transactivation of the pro-inflammatory transcription factor NF-κB (Heiss, Herhaus, Klimo, Bartsch, & Gerhäuser, 2001).

## 3 Cancer chemoprevention in animals by sulforaphane

Sulforaphane (and in a few cases broccoli sprout extracts) have been evaluated as inhibitors of experimental carcinogenesis driven by exposures to chemical or physical carcinogens or genetic mutations. Protective efficacy has been observed following administration of sulforaphane during either the initiation or the post-initiation stages of carcinogenesis. The initial report of the cancer chemopreventive efficacy of sulforaphane was in a model of mammary tumor development in female Sprague-Dawley rats treated with a single dose of the carcinogen 7, 12-dimethyl-benzanthracene (DMBA) (Zhang, Kensler, Cho, Posner, & Talalay, 1994). In this study, after administration of sulforaphane by gavage (75 or 150 μmol per day for 5 days) surrounding the time of exposure to DMBA (and a period of rapid proliferation of mammary epithelial cells), the incidence, multiplicity, and weight of mammary tumors were significantly reduced, and their development was delayed. This model was used later for evaluation of the anti-carcinogenic action of an extract of 3-day old broccoli sprouts, which contains the precursor of sulforaphane, glucoraphanin. Consistent with the findings of sulforaphane, the extract of broccoli sprouts markedly reduced the incidence and multiplicity of mammary tumors (Fahey et al., 1997). A pharmacodynamic study in Sprague Dawley rats demonstrated that sulforaphane could induce NQO1 transcripts, protein and activity to a substantive degree in the mammary epithelium (Cornblatt et al., 2007), consistent with the role of NRF2 in its protective action. Strong pharmacodynamic action reflecting induction of NRF2 target genes was also observed in a bladder cancer inhibition study in rats using lyophilized broccoli sprout extract of known isothiocyanate content (Munday et al., 2008). A recently developed NRF2 knockout rat (Priestley et al., 2016, Taguchi et al., 2016) will allow direct study of the role of the NRF2-sulforaphane connection in these models as wells as those of NRF2 in many other physiological and pathological states.

Studies in murine models (summarized in Table 1) provide evidence for the efficacy of sulforaphane across stages of carcinogenesis. The importance of NRF2 as a target for the actions of several classes of chemopreventive agents, including sulforaphane, was established in a series of studies conducted in wild-type and NRF2 knockout mice (Kensler et al., 2007, Ramos-Gomez et al., 2001). For example, sulforaphane effectively reduced tumor multiplicity of benzo[*a*]pyrene-evoked forestomach tumors in wild-type, but not NRF2-disrupted mice (Fahey et al., 2002). In the classic two-stage mouse skin carcinogenesis model, by which tumors are initiated by DMBA and promoted by repeated dosing with 12- *O*-tetradecanoylphorbol-13-acetate (TPA), sulforaphane inhibited incidence and multiplicity of tumors during the promotion stage (Gills et al., 2006). Xu et al. (2006) observed that pre-treatment with sulforaphane prior to initiation with DMBA and subsequent promotion with TPA reduces the incidence of skin tumors, when compared with the vehicle-pretreated group. Interestingly, no chemoprotective effect was observed with sulforaphane pre-treatment when NRF2-knockout mice were used. This result supports the concept that the KEAP1-NRF2 pathway plays an essential role in the mechanism of action of sulforaphane against skin cancer. Sulforaphane protects wild-type mice against oral cancer induced by treatment with 4NQO (4-nitroquinoline-1-oxide). Parallel studies by another group using the same model demonstrated that sensitivity to oral carcinogenesis was enhanced in NRF2 knockout mice, whilst tumor burden was diminished in KEAP1 knockdown mice. In SKH-1 hairless, high-risk mice, ultraviolet (UV)-radiation-induced skin carcinogenesis was substantially inhibited by topical administration of a broccoli sprout extract containing 1 μmol sulforaphane (corresponding to ca. 50 nmol/cm^2^): incidence and multiplicity were reduced by 50% in the treatment group compared with controls (Dinkova-Kostova et al., 2006). Feeding broccoli sprout extracts providing daily doses of 10 μmol of glucoraphanin was also protective in this model (Dinkova-Kostova et al., 2010). Also in SKH-1 hairless mice, sulforaphane treatment effectively reduced the multiplicity and tumor burden of cutaneous squamous cell carcinomas induced by UVB exposure (Dickinson et al., 2009). Knatko, Higgins, Fahey, & Dinkova-Kostova (2016) found that the incidence, multiplicity and burden of squamous cell carcinomas that form when *Nrf2* is knocked out in KEAP1 knockdown mice [*Keap1(flox/flox)/Nrf2*(−/−)] are much greater than in their *Keap1(flox/flox)/Nrf2(*+/+*)* counterparts, establishing NRF2 activation as the protection mediator.

**Table 1:** Chemopreventive Activity of sulforaphane in mice: Modulation by Nrf2.

Organ site	Species strain	Carcinogen/mutation	SFN Formulation or dose	Endpoints measured	Reference
***Wild-type vs. NRF2-knockout mice***					
Skin	Mouse ♀ C57Bl6	DMBA	100 nmol SFN, topical, q.d. X 14 before DMBA	Reduced tumor incidence in WT with SFN, but not NRF2 KO mice	Xu et al., 2006
Stomach	Mouse ♀ C57Bl/6	4NQO	7.5 μmol SFN q.d. X 9 before/after B[*a*]P	Reduced tumor incidence in WT with SFN, but not NRF2 KO mice	Fahey et al., 2002
Colon	Mouse ♀ C57Bl/6J	4NQO	400 ppm SFN in the diet	Reduced tumor multiplicity and burden in WT mice with SFN; NRF2+/- mice *less* sensitive than WT mice & no protection by SFN	Rajendran et al., 2015
Oral	Mouse ♀ C57Bl/6	4NQO	6 μmol SFN/mouse; 3X wk, p.o. for 16 wk	SFN protects WT at 24 wks	Bauman et al., 2016
	Mouse ♀ C57Bl/6J	4NQO	No treatment	NRF2 KO more sensitive: KEAP1-KD more resistant than WT at 24 wks	Ohkoshi et al., 2013
Skin	Mouse ♀ SKH-1	UV	100 μL broccoli sprout extract containing 1 μmol SFN topical	Reduced tumor incidence, multiplicity & burden	Dinkova-Kostova et al., 2006
	Mouse ♀ SKH-1	UV	broccoli sprout extract providing 10 μmol glucoraphanin daily in the diet	Reduced tumor incidence, multiplicity & burden	Dinkova-Kostova et al., 2010
	Mouse ♀ SKH-1	UV	No treatment	NRF2 KO much more sensitive than KEAP1-KD	Knatko et al., 2016
***Wild-type rodents only***					
Skin	Mouse ♀ CD-1	DMBA→ TPA	1, 5 or 10 μmol SFN topical before TPA	Reduced tumor incidence & multiplicity	Gills et al., 2006
Skin	Mouse ♀ SKH-1	UV	2.5 μmol SFN topical	Reduced tumor incidence & multiplicity	Dickinson et al., 2009
Colon	Mouse ♂ C57Bl/6J^+/min^	Apc^min^	∼6 μmol SFN/d (443 ppm) in diet for wks 6–16	Reduced tumor multiplicity	Myzak et al., 2006
Colon	Mouse	Apc^min^	300 or 600 ppm SFN in diet for wks 8–11	Dose-dependent reduction in tumor multiplicity	Hu et al., 2006
Colon	Mouse ♂	Apc^min^	600 ppm SFN in diet for wks 5–15	Reduced tumor multiplicity	Shen et al., 2007
Lung	Mouse ♀ A/J	B[*a*]P + NNK	3 mmol/kg; 20 wks after carcinogen administration, fed diet containing SFN wks 21–42.	Reduced tumor incidence	Conaway et al., 2005
Prostate	Mouse ♂	TRAMP	6 μmol SFN/mouse; 3X wk, p.o. for 17–19 wk	Reduced tumor incidence	Singh et al., 2009
Prostate	Rat ♂	TRAMP	60 and 240 mg broccoli sprouts/mouse/day, p.o., for 16 wk	Reduced tumor incidence	Keum et al., 2009
Bladder	Rat ♀	BBN	lyophilized broccoli sprout extract in diet to provide isothiocyanate doses of 40 and 160 μmol/kg body weight/d	Reduced tumor incidence, multiplicity and size	Munday et al., 2008

**Abbreviations:** KO, knockout, KD, knockdown; WT, wild-type; SFN, sulforaphane; DMBA, dimthylbenz[a]anthracene; TPA, 12-O-tetradecanoylphorble ester; 4NQO, 4-nitroqquinoline-1-oxide); UV, ultraviolet light; B[a]P, benzo[a]pyrene; AOM, azoxymethane; DMH, dimethylhydrazine; N-OH-BBN, N-butyl-N-(4-hydroxybutyl) nitrosamine; TRAMP, transgenic adenocarcinoma of mouse prostate; NNK: 4-(methylnitrosamino)-1-(3-pyridyl)–1-butanone.

Another informative model is the Apc^min^ (adenomatosis polyposis coli; multiple intestinal neoplasia) mouse, in which a transversion point mutation introduces a stop codon that leads to an increased burden of intestinal tumors. Several groups have shown that treatment of Apc^min^ mice beginning weeks to months after birth with dietary sulforaphane provoked substantial reductions in tumor multiplicity and overall tumor burden (Hu et al., 2006, Myzak et al., 2006, Shen et al., 2007). To date, no studies have been conducted to examine the effects of NRF2 genotype on tumor outcomes in this model or upon the protective actions of sulforaphane.

## 4 A “dark” side to NRF2 signaling

Although activation of NRF2 signaling is generally regarded as cytoprotective, and hence a useful target for prevention of cancer and other diseases, cancer genome sequencing efforts have indicated a substantial representation of mutations in the interaction domains of KEAP1 and NRF2 that lead to constitutive activation of NRF2 signaling in cancer cells (Hayes and Dinkova-Kostova, 2014, Praslicka et al., 2016). Thus, cancer cells frequently highjack the pathway to promote their survival and growth. These actions have led to controversy whether activation, or alternatively inhibition, of NRF2 are useful strategies for the prevention or treatment of cancer. As thoughtfully addressed by Sporn and Liby (Sporn & Liby, 2012), the answers lie within the context of the specific opportunities. In genetic models of pathway disruption or hyper-activation, the “dose-response” curve is often “U”-shaped (Kensler & Wakabayashi, 2010); pharmacological or nutritional modulation occurs in a limited dynamic range in the middle ground. Thus, the genetic models with constitutive activation (or loss) of NRF2 signaling are poor mimetics or predictors of the actions of small molecule-based inducers of the pathway where change in signaling activity is reversible and intermittent. Situations where chronic pharmacological interventions with potent activators of NRF2 fail to phenocopy the effects of genetic constitutive activation highlight this point. With regards to sulforaphane, there is certainly a continuing need for the monitoring of safety with long-term administration in pre-clinical models as well as clinical trials. Nonetheless, prolonged treatment with sulforaphane did not enhance tumorigenesis in oncogenic K-ras and xenograft mouse models of lung cancer (Kombairaju et al., 2011), whilst genetic modulation of NRF2 state has been shown to affect lung carcinoma development (Jeong et al., 2017, Satoh et al., 2013).

## 5 Demonstrating pharmacodynamic action of sulforaphane in humans: NRF2 signaling as a probe

There is considerable interest in developing small molecules that activate the NRF2 signaling pathway in humans for prevention and treatment of multiple acute and chronic diseases. One such drug, tecfidera (dimethylfumarate) was approved by the FDA in 2012 for treatment of relapsing multiple sclerosis. The oleanane triterpenoid bardoxolone methyl is used in clinical trials for treatment of chronic kidney disease and pulmonary arterial hypertension. ClinicalTrials.gov lists over a score of trials using “broccoli” or “sulforaphane” in multiple disease settings including asthma, autism, schizophrenia, cystic fibrosis, sickle cell, alcohol intolerance, cardiovascular disease, immune response to influenza, dermatitis and cancer prevention. Published results from such trials were reviewed in 2015 (Conzatti, Fróes, Schweigert Perry, & Souza, 2015).

Among many challenges in the design and implementation of such trials are the selection of an adequate dose, type of formulation and dose schedule. Biomarkers, tools for the assessment of pharmacodynamic action of sulforaphane, are extremely useful in this regard. While there has been considerable progress in characterizing the pharmacokinetics of various broccoli/sulforaphane preparations (Atwell et al., 2015a, Atwell et al., 2015b, Egner et al., 2011, Fahey et al., 2012b) and improved formulations with which to provide more consistent bioavailability (Fahey et al., 2015), there is limited evidence for target modulation in humans, be it the putative target NRF2 or something else. Three general approaches have been utilized. 1. Examination of the serum secretome; 2. gene expression changes in peripheral blood mononuclear cells or other accessible cells (e.g., skin or buccal cells or nasal swabs); and 3. altered “drug” metabolism phenotypes. There have now been many clinical studies that have utilized either orally or topically (skin surface) delivered sulforaphane (Table 2). Delivery vehicles range from fresh broccoli or broccoli sprouts to commercial nutritional supplements containing glucoraphanin or stabilized sulforaphane, to custom preparations that are highly enriched in these phytochemicals but may never be suitable for large populations. Many of the studies have used a dried extract of broccoli sprouts or seeds. Unfortunately, whereas many clinical studies have chemically characterized the preparations used, and have used standardized preparations (reviewed by: Fahey and Kensler, 2007, Fahey et al., 2012a), others have not. In addition to purely pharmacokinetic evaluations which we do not address herein, many of the clinical studies summarized in Table 2 have gone beyond KEAP1-NRF2-ARE related outcomes and examined outcomes or biomarkers related to some of sulforaphane's other modes of action (e.g. antibiosis, anti-inflammatory). These may or may not involve cross-talk with the NRF2 pathway and thus may be of interest in the context of this review.

**Table 2:** Modulation of NRF2 targets in clinical studies with broccoli preparations.

Agent	Dose and Schedule	Sample Size (duration)	aBiomarker Modulation	References
**Studies demonstrating an NRF2-related pharmacodynamic effect**				
Broccoli Sprout Beverage (GRR)	●7Placebo, q.d. ●400 μmol GRR q.d.	200 (14 days)	9% decrease in urinary excretion of AFB-N7-gua DNA adducts at 10 days; 10% decrease in pollutant PheT excretion	Kensler et al., 2005
Broccoli Sprout Extract (SFR)	●5, 40, 170 or 340 nmol sulforaphane-rich BSE applied topically once	17 (1 dose)	Increased NQO1 activity (> 1.5-fold) in skin punch biopsies 24 h after topical application of 170 or 340 nmol SFN containing BSE	Dinkova-Kostova et al., 2007
Broccoli Sprout Extract (SFR)	●50, 100, 150, or 200 nmol sulforaphane-rich BSE applied topically, 3 times	17 (3 doses, every 24 h)	Dose-dependent increase in NQO1 activity (up to 4.5-fold) in skin punch biopsies 24 h after the last dose	Dinkova-Kostova et al., 2007
Broccoli Sprout Extract (SFR)	●200 or 400 nmol sulforaphane-rich BSE applied topically, 3 times	6 (3 doses, every 24 h)	↓erythema (by ∼40%) on 5^th^ day, from narrow band UVB (340 nm) irradiation on 4^th^ day, following SF- compared to solvent-treatment	Talalay et al., 2007
Broccoli Sprout Homogenate (SFR)	●25, 50, 75, 100, 125, 150, 175, 200 g broccoli sprout homogenate (BSH) ●200 g alfalfa sprout homogenate (ASH) q.d.	57 (3 days)	Doubling (*GSTP*) or tripling (*NQO1*) of gene transcripts in nasal lavage after 3 doses of 200 g (102 μmol) BSH but not ASH.	Riedl et al., 2009
Broccoli Sprout Homogenate (SFR)	●200 g broccoli sprout homogenate	12 (3 days)	Significant increase in protein levels of secretory leukocyte protease inhibitor in nasal lavage after 48 h.	Meyer et al., 2013
Broccoli Sprout Beverage (GRR ↔ SFR) Cross-over	●Run-in → GRR (800 μmol) → wash-out → SFR (150 μmol) ●Run-in → SFR → wash-out → GRR	50 (24 days)	20–50% increases in urinary excretion of mercapturic acid conjugates of air pollutants: acrolein, ethylene oxide, crotonaldehyde, benzene	Kensler et al., 2012
Broccoli Sprout Beverage GRR + SFR Blend	●Placebo ●GRR (600 μmol) + ●SFR (40 μmol)	291 (84 days)	Rapid and sustained increases in the rate of urinary elimination of mercapturic acids of benzene (61%) and acrolein (23%), but not crotonaldehyde	Egner et al., 2014
Broccoli Sprout Homogenate (SFR)	●SFR (100 μmol)	45 (14 days)	Positive association between increased FEV_1_ response to methylcholine and induction of *GCLM* and *NQO1* transcripts in peripheral blood mononuclear cells in response to sulforaphane.	Brown et al., 2015
Broccoli Sprout Extract (BSE) (GRR capsule)	●Placebo ●BSE capsules [10 mg GR ea. (23 μmol)] 3 capsules q.d.	52 (96 days)	Significant reduction in urinary excretion of 8-OHdG compared to placebo.	Kikuchi et al., 2015
Broccoli Sprout Extract (SFR)	●Single (200 μmol) & dual (100 μmol, q12h)	20 (1 day)	No induction of HO-1 observed; transient decrease in HDAC activity observed at 3 h post dosing	Atwell et al., 2015a
Broccoli Sprout Extract (BSE) (GRR capsule)	●BSE capsules [10 mg GR ea. (23 μmol)] 3 or 6 capsules q.d.	21 (3 days)	Dose dependent increases in serum enzyme activities of GST (CDNB) and NQO1.	Ushida et al., 2015
Broccoli Sprout Beverages (GRR or SFR)	●Single arm crossover GRR (600 μmol) → SFR (40 μmol)	10 (5 days)	Induction of *NQO1* transcripts in buccal cells scraped from inner cheek with GRR or SFR beverages compared to run-in.	Bauman et al., 2016
Broccoli Sprout Homogenate (SFR)	●50–150 μmol dose escalation	14 (21 day trt + 28 day washout)	Increase in whole blood mRNA for HMOX1 and trend for same with HBG1 but no sig Δ in HbF, in sickle cell disease (SCD) patients.	Doss et al., 2016
Broccoli Sprout Homogenate	●200 g BSH ●Placebo = 200 g alfalfa sprout homogenate	15 (3 days)	No increased expression of NRF2-regulated gene transcripts (GSTM1, HO-1, NQO1, NRF2) in nasal epithelial cells or peripheral blood. No decrease in %PMNs in sputum following O3 challenge.	Duran et al., 2016
**Studies demonstrating an effect that is not [necessarily] NRF2-related**				
Fresh BS	●100 g fresh wt. BS (∼600 μmol GR)	12 (7 days)	↓PCOOH, ↓8OHdG, ↓8iso, ↑CoQ, ↑HDL-C (♀ only)	Murashima et al., 2005
Fresh BS	●318–1271 μmol GR	9 (7 days)	Subjects – *H. pylori* infected: 7 of 9 appeared “cured”; between 2 and 6 still cured after 35 days	Galan et al., 2004
Cooked B (Hi- and Low-GR soup)	●344 and 102 μmol GR	16 (1 day /single dose)	↑regulation of various genes involved in xenobiotic metabolism, including those assoc. with NRF2 pathway (e.g. AKR, GCLM) and the heat shock pathway	Gasper et al., 2007
Fresh BS	●68 g BS (∼593 μmol SF)	3 (21 days)	↓HDAC in PBMCs and ↑acetylated histones H3 & H4 at 3 & 6 h post consumption	Myzak et al., 2007
Fresh BS or BS supplements	●68 g BS or 6 pills of supplement (∼3 g of freeze dried BS)	24 (7 days)	↓HDAC in PBMCs at 12 and 48 h after the final dose of sprouts or supplement	Clarke et al., 2011
Steamed B	●Placebo (400 g peas per week) ●400 g B per week	22 (1 year)	Δ in mRNA processing, TGFβ1, IL-2, NOTCH, WNT, EGFR1, and insulin signaling in prostate needle biopsies	Traka et al., 2008
Fresh BS	●Placebo ●420 μmol GR	50 (54 days)	Subjects – *H. pylori* infected: Considerable ↓*H. pylori* infection, and ↓pro-inflammatory markers, ↓UBT, but no complete eradication	Yanaka et al., 2009
BSE (GR)	●200 μmol GR-rich BSE, orally	4 (single dose)	↓inactivation (by >95%) of macrophage migration inhibitory factor (MIF) tautomerase activity in urine 8 h after dosing	Healy et al., 2011
BSP (SFR)	●placebo ●112 μmol SF^b^ ●224 μmol SF^b^	81 (28 days)	Subjects - with type 2 diabetes: ↓inflammatory markers in high SF group compared with placebo ↓fasting glucose, total cholesterol & LDL levels in both groups: no effect on insulin sensitivity ↓malondialdehyde	Mirmirin et al., 2012 Bahadoran et al., 2011, Bahadoran et al., 2012a, Bahadoran et al., 2012b
Blanched, Frozen B	●Placebo (peas) ●High GR B (21.6 μmol/g dry wt.) ●Low GR B (6.9 μmol/g dry wt.)	48 (84 day)	Biomarkers of CVD risk; ↓variation in lipid and a.a. metabolites and TCA cycle intermediates suggesting altered control points	Armah et al., 2013
BSE (SFR)	450 μmol SF/day delivered in cheese-based soup ●SF, ●SF+RIF, ●RIF (rifampicin)	24 (7 days x 3)	Subjects – healthy volunteers characterized for CYP3A4 status: SF treatment did not affect CYP3A4 activity	Poulton et al., 2013
BSP (SFR)	●Standard triple therapy ●BSP (135 μmol SF/d)^b^ ●BSP + triple therapy	86 (28 days)	Subjects – type 2 diabetes / *H. pylori* infected patients: Considerable ↓*H. pylori* infection, and ↓pro-inflammatory markers, but no complete eradication	Bahadoran et al., 2014
BSE (SFR)	●100 μmol SF/d delivered in mango juice	29 (4 days BSE trtmnt)	Subjects challenged with an irritation/allergy-provoking diesel exhaust particle (DEP) suspension; white blood cell counts declined by 54% when DEP challenge was preceded by daily BSE admin for 4 days	Heber et al., 2014a, Heber et al., 2014b
BSE (SFR)	●200 μmol SF/d	20 (≤140 days)	Subjects – men with biochemical recurrence of prostate cancer: PSA doubling time was 9.6 mo. on-trtmnt, vs. 6.1 mo. pre-trtmnt; 1 subj had >50% ↓PSA; 7 subj had ≤50% declines; (no placebo group)	Alumkal et al., 2015
B (Blanched, Frozen)	●High GR B (21.6 μmol GR/g dry wt.) ●Low GR B (6.9 μmol GR/g dry wt.)	37 (84 days)	Subjects - w/ elevated CVD risk: Measured blood lipid markers in; Found ↓LDL-C w/ High GR B No sig diff in TC, HDL-C, TAG	[Study 1] Armah et al., 2015 [Study 2]
	●High GR B (24.8 μmol GR/g dry wt.) ●Low GR B (9.5 μmol GR/g dry wt.)	96 (84 days)		
BSdE (GRR)	2 pills, 3x/d: ●placebo ●514 μmol GR/d	54 (56 days)	Subjects - breast biopsy candidates: ↓Ki67, ↓HDAC3 in benign tissue, ↓HDAC in PBMCs	Atwell et al., 2015b
BSdE (SFR)	●placebo ●339 μmol SF/day in tablets for 6 mo., followed by 2 mo. non treated follow-up	78 (182 days)	Subjects – radical prostatectomy patients: PSA doubling time was 86% longer in SF than placebo group (28.9 & 15.5 months respectively). SF effects prominent at 3 mo. and maintained throughout	Cipolla et al., 2015
BSE (SFR)	●200 μmol SF-rich, or ●200 μmol GR-rich BSE, applied topically daily × 3 d	24 (5 days)	↓erythema on 5^th^ day, from solar simulated UV irradiation on 4^th^ day, following SF- but not GR-treatment	Knatko et al., 2015
BSdE (GR)	Daily, 3 oral tablets delivering: ●69 μmol GR	10 (54 days)	Subjects - shizophrenia outpatients: PANSS & CGI (cognitive function tests) showed suggestion of improvement; serum BDNF (nsd)	Shiina et al., 2015
BSE (SFR)	●50 μmol SF ●100 μmol SF ●200 μmol SF	17 (28 days)	Subjects – w/ melanoma & multiple atypical/dysplastic nevi: Δs in pSTAT3 (nsd); ↓pro-inflamm factors (nsd); & ↓tumor suppressor decorin	Kirkwood et al., 2016
Fresh BS	●Placebo ●100 g BS	40 (3 days)	Subjects – asthmatics w/ pos. skin test to indoor allergen: No differential effect on asthma-related endpoints including NRF2-related; no measurement of actual dose (SF or GR)	Sudini et al., 2016
Fresh BSH	●Placebo ●100 μmol SF	29 (21 days)	Subjects innoc. w/ FluMist LAIV (Live Attenuated Influenza Virus): ↑peripheral blood NK cell expression (granzyme B production) & ↓circulating influenza RNA	Müller et al., 2016

**Abbreviations:** 8iso, 8-isoprostane; 8OHdG, 8-hydroxy 2′-deoxy guanosine; AFB-N7-gua, aflatoxin B1-N7-guanine; ASH, alfalfa sprout homogenate; B, broccoli; BS, broccoli sprouts; BSdE, broccoli seed extract; BSE, broccoli sprout extract; BSH, broccoli sprout homogenate; BSP, broccoli sprout powder; CoQ, CoQ_10_H_2_/CoQ_10_ ratio; CVD, cardiovascular disease; GR, glucoraphanin; GRR, glucoraphanin-rich; HDL-C, High Density Lipoprotein – Cholesterol; LDL-C, Low Density Lipoprotein – Cholesterol; nsd, no significant difference; PANSS, positive and Negative syndrome scale; PCOOH, phosphatidylcholinyl hydroperoxide; PheT, phenanthrene tetraol; SFN, sulforaphane; SFR, sulforaphane-rich; TAG, triglycerides; TC, total cholesterol; TTR, transthyretin; ZAG, zinc α-2 glycoprotein.

a Subjects were healthy unless otherwise indicated at the beginning of these sections.

b Nominally designated at SF-rich, but it is clear that the SF titer of these powders is not as advertised and the investigators did not do further analysis of GR or SF titer.

Feeding studies with cruciferous vegetables, presumably rich in isothiocyanates, have demonstrated increased circulating levels of NRF2 target gene products (e.g., GST, NQO1), measured as proteins or enzymatic activities. Bogaards (Bogaards, Verhagen, Willems, van Poppel, & van Bladdern, 1994) reported in a clinical study, small but significant increases in plasma levels of α-class GST were observed in volunteers consuming a diet enriched in Brussels sprouts. Navarro (Navarro et al., 2009) similarly demonstrated modulation of human serum GSTA1/2 concentration by cruciferous vegetables in a controlled feeding study. Sreerama (Sreerama, Hedge, & Sladek, 1995) reported increased enzymatic activity of GSTs and NQO1 in the saliva of subjects who continually ingested large quantities of broccoli. In no cases were content of sulforaphane or other isothiocyanates measured in the dietary vegetables used. Other proteins show larger dynamic range of induction through NRF2 activation (e.g., AKRs); while increased concentrations have been observed in the media of cells following treatment with sulforaphane (Agyeman et al., 2012), they have not been examined in clinical samples. Increased annotation of the human serum secretome coupled with deeper interrogation with new mass spectrometric methods offers prospects for the identification of secreted, circulating proteins reflecting the pharmacodynamic action of sulforaphane in clinical trial settings. In a similar vein, metabolomics surveys of biofluids may provide candidate markers exhibiting sufficient abundance, specificity and dynamic range in response to changes in signaling flux through the NRF2 pathway.

Elevated levels of gene transcripts for NRF2 target genes such as *NQO1* and *GSTs* have been reported in healthy volunteers following administration of broccoli-based glucoraphanin/sulforaphane preparations in skin punch biopsies, nasal scrapings, buccal scrapings, peripheral blood mononuclear cells, and whole blood collections for isolation of mRNA (Bauman et al., 2016, Brown et al., 2015, Doss et al., 2016, Dinkova-Kostova et al., 2007, Riedl et al., 2009). These studies signal the likely activation of the NRF2 pathway in these cell types, but optimization of dose, formulation, timeframe and tissue processing have not been undertaken rigorously to date. Additionally, studies to link the magnitude of change in expression of marker genes with more functional endpoints have not been conducted.

Pharmacologic manipulations and crucifer-rich diets have been shown to modify the “phase 2” or conjugation metabolism of antipyrine, phenacetin, oxazepam, and acetaminophen in humans (Pantuck et al., 1979, Park and Kitteringham, 1990). Rather than using drugs to monitor phenotypic changes in metabolic pathways as done in these early studies, we have relied – and in fact targeted – environmental exposures to food and airborne carcinogens with the purposeful intention of increasing rates of their detoxication with broccoli-based interventions.

In a 2009 cross-over clinical trial conducted in Qidong, China, in which 50 healthy subjects were recruited to take two broccoli sprout-derived beverages: one glucoraphanin-rich (GRR) and the other sulforaphane-rich (SFR), the pharmacodynamic actions of these two beverages were compared (Kensler et al., 2012). Urinary excretion of the mercapturic acids of the air-borne toxins acrolein, crotonaldehyde, ethylene oxide, and benzene were measured in urine samples from both pre- and post-interventions using liquid chromatography tandem mass spectrometry. Statistically significant increases of 20%–50% in the levels of excretion of glutathione-derived conjugates of acrolein, crotonaldehyde and benzene were seen in individuals receiving SFR, GRR, or both compared with their pre-intervention baseline values. No significant differences were seen between the effects of SFR versus GRR on the pollutant biomarker levels. In a more recent 12-week placebo-controlled, randomized clinical trial, in which 291 participants from Qidong were provided a broccoli sprout beverage containing both 40 μmol sulforaphane and 600 μmol glucoraphanin, the urinary levels of the mercapturic acids of the air pollutants, benzene, and acrolein were measured and used as biomarkers of health risk. The detoxification of these airborne pollutants was enhanced by the broccoli sprouts beverage. The levels of excretion of the glutathione-derived conjugates of benzene (61%) and acrolein (23%) were significantly higher in the participants who received the broccoli sprout beverage compared with placebo. This increase in pollutant-mercapturic acid excretion was rapid and sustained throughout the intervention (Chen et al., 2012). Overall, this study provided strong evidence that broccoli sprout beverage can modulate the disposition of environmental carcinogens and toxins. The role of NRF2 in these actions is not established but inferred as influences of polymorphisms in GST isoforms and in the promoter region of NRF2 itself on the rates of detoxication of benzene were noted.

## 6 KEAP1 and done?

By no means is this so. Many complex diseases have proven historically to be resistant to mono-preventive or therapeutic approaches. Mechanisms for resistance can be many-fold, and in addition to factors affecting the pharmacokinetics of the molecule, loss or alteration in the primary molecular target can become a substantive barrier to efficacy. As highlighted in this review, there is ample evidence that sulforaphane, administered in a variety of broccoli-based formulations – or as pure compound to animals – can activate the KEAP1-NRF2 signaling pathway. What is less clear is whether this pathway is the primary target (i.e., preferentially affected at the lowest concentrations). Very few dose-response studies have been conducted in humans, animals, or even cell culture systems that provide much guidance of the hierarchy of sulforaphane interactions with cysteine-rich targets, be it KEAP1 or other proteins (or indeed that cysteine residues are the only chemical targets, e.g., lysine). A potential attraction for the use of sulforaphane, in addition to the profound feasibility of developing practical, effective broccoli-based formulations for administration, is the possibility that multiple pathways are perturbed and that *in toto*, such actions provide stronger opportunities for disease prevention or treatment. As exemplified in the studies listed in Table 2, sulforaphane can modulate other signaling pathways and biological processes underlying the etiopathogenesis of complex disease states. Continued evaluation of the pharmacokinetics and pharmacodynamic action by tracking the actions of sulforaphane on the KEAP1-NRF2 stress response system provides one means to optimize the development of intervention strategies and to match the intervention to the appropriate at-risk populations. However, it is only one guidepost on the trail to effective, frugal disease mitigation.

## Funding

This work was supported by the 10.13039/100000002National Institutes of Health [R35 CA197222, P50 CA097190], The Lewis B. and Dorothy Cullman Foundation, 10.13039/501100000289Cancer Research UK [C20953/A18644], and 10.13039/501100000268BBSRC [BB/L01923X/1].
